# Freezing biological organisms for biomedical applications

**DOI:** 10.1002/SMMD.20220034

**Published:** 2022-12-27

**Authors:** Gaizhen Kuang, Qingfei Zhang, Jinxuan Jia, Yunru Yu

**Affiliations:** ^1^ Pharmaceutical Sciences Laboratory Åbo Akademi University Turku Finland; ^2^ Oujiang Laboratory (Zhejiang Lab for Regenerative Medicine, Vision and Brain Health) Wenzhou Institute University of Chinese Academy of Sciences Wenzhou China

**Keywords:** biological organisms, cryoablation, cryopreservation, tissue regeneration, transplantation

## Abstract

Biological organisms play important roles in human health, either in a commensal or pathogenic manner. Harnessing inactivated organisms or living organisms is a promising way to treat diseases. As two types of freezing, cryoablation makes it simple to inactivate organisms that must be in a non‐pathogenic state when needed, while cryopreservation is a facile way to address the problem of long‐term storage challenged by living organism‐based therapy. In this review, we present the latest studies of freezing biological organisms for biomedical applications. To begin with, the freezing strategies of cryoablation and cryopreservation, as well as their corresponding technical essentials, are illustrated. Besides, biomedical applications of freezing biological organisms are presented, including transplantation, tissue regeneration, anti‐infection therapy, and anti‐tumor therapy. The challenges and prospects of freezing living organisms for biomedical applications are well discussed. We believe that the freezing method will provide a potential direction for the standardization and commercialization of inactivated or living organism‐based therapeutic systems, and promote the clinical application of organism‐based therapy.

## Graphical abstract

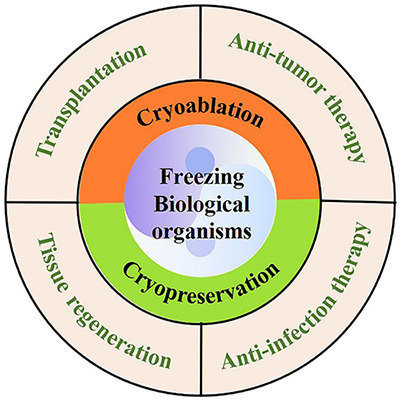

1

**Key points**

- We introduce the freezing strategies of cryoablation and cryopreservation.
- We present the biomedical applications of freezing biological organisms.
- This review will provide guidance for utilizing freezing methods to achieve biological organism‐based therapies.

## 2 INTRODUCTION

Biological organisms have varied influences on human beings, some of which are symbiotic with humans while others may cause diseases.^1^ With the development of biology and progress in material science, biological organism‐based therapy has been an important branch of biological medicine. For instance, fecal microbiota transplantation has been widely utilized in gastrointestinal diseases.^2^ Besides, cell‐based therapies are developed rapidly and show great application prospects in tissue engineering and cancer treatment.^3^ Cells with proliferation and differentiation abilities (mainly stem cells) can repair or replace tissues in damaged or pathological conditions.^4^ Engineered immune cells (such as chimeric antigen receptor T‐cell [CART] and dendritic cells [DC] vaccine) have shown potential in cancer treatment.^5^ In addition to the direct use of biological organisms for biomedical applications, the inactivation of detrimental organisms is also an important research direction. The inactivated tumor cells by radiation or chemical reagents can act as cancer vaccines.^6^ Although with many successes, a simpler approach is needed for the inactivation of tumor cells. Besides, therapies using living organisms encounter the challenge of effective long‐term storage and high viability when applied, which greatly impedes further clinical translation. Therefore, a facile regime is highly anticipated for both inactivated organism‐based therapy and living organism‐based therapy.

With the in‐depth study of cryobiology, the effects of low temperatures on biological organisms are becoming increasingly clear. Initial studies suggested that living organisms left at −20°C for 1 min will cause necrosis.^7^ Subsequently, the importance of rapid freezing, slow thawing, and freeze‐thaw cycles was recognized.^8^ Cryoablation causes cellular damage, death, and necrosis of tissues by direct mechanisms, which cause cold‐induced injury to cells, and indirect mechanisms, which cause changes to the cellular microenvironment and impair tissue viability. Nowadays, cryoablation has been widely studied in the treatment of cancer, atrial fibrillation, dermatoses, neuralgia, and so on.^9^ Compared to cryoablation, which inactivates living organisms by freezing method, cryopreservation can store living organisms at a very low temperature.^10^ Two key factors in the success of cryopreservation are cryoprotective agents (CPAs) and temperature control. CPAs make it possible to store cells at cryogenic temperatures and recover cell viability and functionality at high levels.^11^ With the improvement of temperature control devices, cryopreservation from cells to organs has become a routine in basic research and biomedical applications. By reasons of the foregoing, it is possible to simply achieve inactivated organism‐based therapy or living organism‐based therapy by the freezing method.

**FIGURE 1:**
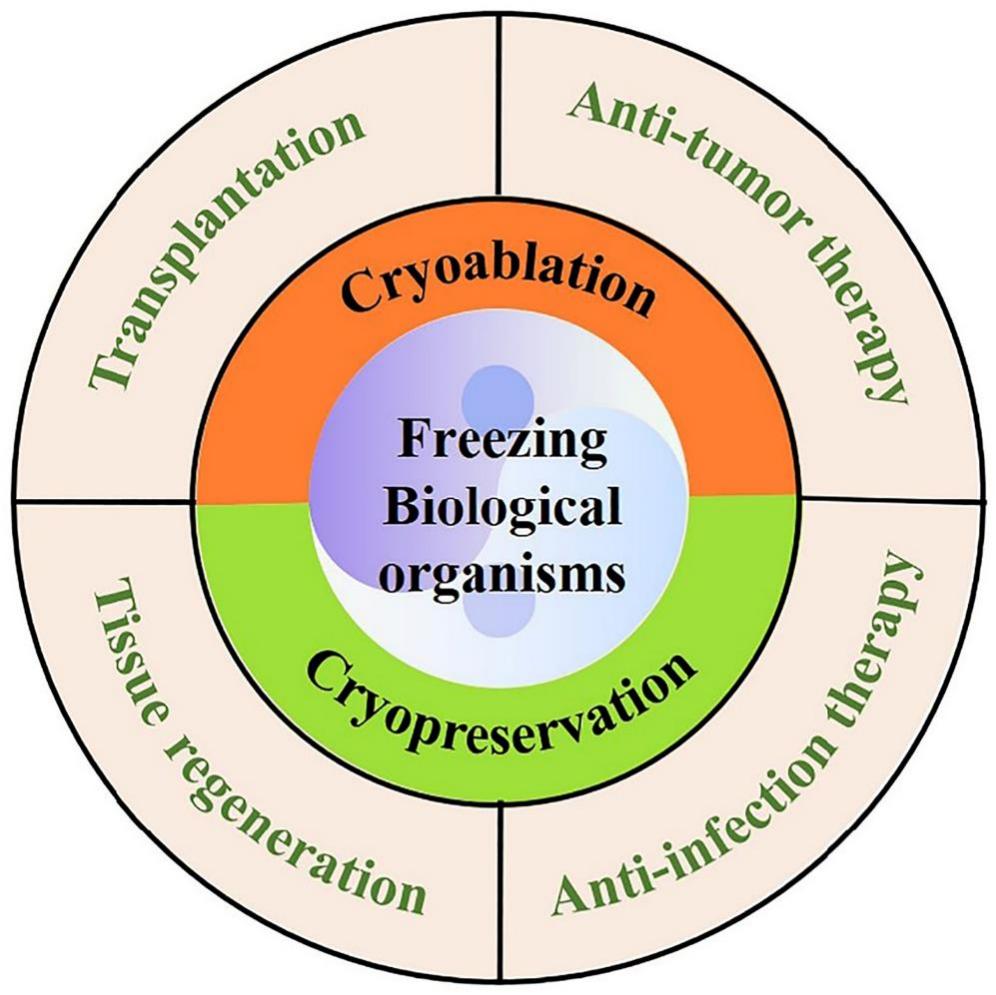
Freezing biological organisms by cryoablation or cryopreservation is widely applied in the fields of transplantation, tissue regeneration, anti‐infection therapy, and anti‐tumor therapy.

In this review, we summarize the advances in freezing biological organisms for biomedical applications (Figure 1). First, we introduce the freezing strategies of cryoablation and cryopreservation. The freezing strategies of cryopreservation include the cryopreservation of free organisms, microencapsulation‐based cell delivery systems, and scaffold‐based cell delivery systems. Then, we survey recent applications of freezing biological organisms in transplantation, tissue regeneration, anti‐infection therapy, and anti‐tumor therapy. Finally, we summarize the merits and challenges of this approach and put forward the prospect of future application. The overall objective of this review is to discuss the biomedical potential of freezing biological organisms, as well as provide guidance for utilizing freezing methods to achieve the inactivated or living organism‐based therapies.

## 3 FREEZING STRATEGIES OF CRYOABLATION

Cryoablation is a method of destroying tissue by freezing. The processes of cryoablation mainly include quick freezing and slow thawing. During these processes, the tissues are damaged by cellular injury and vascular injury.^8^ Due to convenient access and visualization of the surgical field, dermatological diseases, including benign neoplasm, precancerous lesions, some malignant tumors, and other superficial tumors such as breast cancer are amenable to cryosurgery treatment.^9^ ^a,^ ^12^ With the development of cryosurgical devices, cryogen, and iconography, cryotherapy is becoming more and more widely used. Compared with traditional surgical methods, cryoablation owns some advantages, such as faster postoperative recovery and less pain. As an attractive alternative to radiofrequency ablation, cryoablation has shown comparable safety and efficacy in the treatment of atrial fibrillation.^9^ ^b^ Besides, cryoablation has attracted renewed interest in neuropathy management and can provide adequate pain relief in several weeks to months.^9^ ^e^ Other than superficial tumors, tumors such as liver, pancreas, and lung malignancies might achieve minimally invasive treatment with the image‐guided intervention by cryoablation.^13^

**FIGURE 2:**
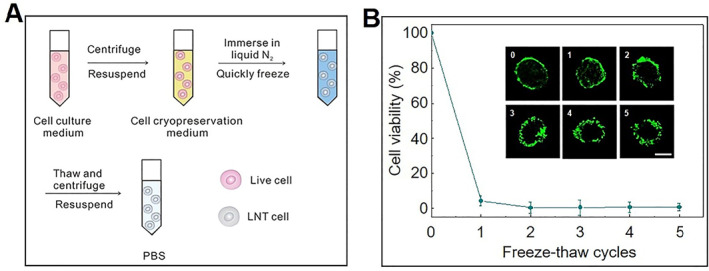
Tumor cell inactivation by the freezing method. (A) Schematic of the procedure to prepare liquid nitrogen‐treated tumor (LNT) cells. Reproduced with permission.^14^ The Authors, published by American Association for the Advancement of Science. (B) Cell viability and fluorescence images of tumor cells treated with different freeze/thaw cycles at −20°C/37°C. Scale bar is 10 μm. Reproduced with permission.^16^ Copyright 2013, Elsevier Ltd.

Similar to the tissue injury caused by cryoablation, inactivated tumor cells by the freezing method as whole cell cancer vaccines have become a new hotspot of immunotherapy. For the number of the freeze‐thaw cycle, the present research studies mainly focus on one or two cycles. Gu et al. immersed the tumor cell suspension into liquid nitrogen for 12 h and then thawed them at 37°C (Figure 2A).^14^ These liquid nitrogen‐treated tumor cells lost viability, proliferative activity, and pathogenicity in vivo while retaining the tumor antigens, which means the cells treated with liquid nitrogen could act as a cancer vaccine. Serda et al. also used one freeze‐thaw cycle, and they transferred the cancer cell‐containing medium to −80°C for 24 h to get whole cell cancer vaccine.^15^ Su et al. studied the viability and integrity of cancer cells treated by different freeze/thaw cycles at −20°C/37°C (Figure 2B). Almost 100% of tumor cells could be inactivated when subjected to two successive free‐thaw cycles.^16^ In conclusion, it is feasible to prepare whole cell cancer vaccines by the freezing method.

## 4 FREEZING STRATEGIES OF CRYOPRESERVATION

Cryopreservation is the process of freezing cells and organs at extremely low temperatures to preserve them.^10^ With the development of cryobiology and the use of CPAs and temperature control equipment, cryopreservation of biological constructs has become a routine. Since the establishment of the cryoprotective effect of glycerol, the family of CPAs is growing and mainly includes cell permeating agents (dimethyl sulfoxide [DMSO], glycerol, ethylene glycol [EG], propylene glycol, etc.), and non‐permeating agents (sucrose, trehalose, polyethylene glycol, polyvinyl pyrrolidone, etc.). Cell permeating agents can enter cells quickly and inhibit the production of intracellular ice crystals, but have different degrees of toxicity on cells. Non‐permeating agents cannot penetrate the cell membrane but they can increase the extracellular osmotic pressure.^11^ The proper selection of CPAs is important to improve the viability of the cryopreserved samples.

According to the cooling rate, cryopreservation can be roughly divided into two methods of slow freezing and vitrification (Table 1). Typically, slow freezing occurs at a cooling rate of 1°C/min in a benchtop portable freezing container or a controlled‐rate freezer. In this process, low‐concentration CPAs (<2M) are used without high contamination risk and manipulation skills. However, slow freezing faces the problem of cryo‐induced injury during the formation of extracellular ice. Different from slow freezing, vitrification requires rapid cooling of the samples into deep cryogenic temperatures with high concentrations (>5M).^17^ By this method, cells or tissues are directly transformed into a glass state without ice nucleation formation. Although with the advantage of avoiding freeze injury, CPA‐induced cytotoxicity, high risk of pathogenic agent contamination, and high demand for manipulation skills need to be considered.^18^

**TABLE 1:** Comparison of slow freezing and vitrification methods

Characteristics	Slow freezing	Vitrification
Cooling rate	Usually −1°C/min from +4°C to −70…−80°C followed by storage in LN_2_	−10^2^…10^4^°C/min by immediate transfer to LN_2_
CPA types	Most commonly contain DMSO, may contain ethylene glycol, glycerol, etc.	Complex cocktails
CPA concentrations	5%–10% (v/v)	>25% (v/v)
Equipment	Benchtop portable freezing container or a controlled‐rate freezer	Equipment with liquid nitrogen
Damage to the object	Mechanical damage (ice formation)	Chemical damage (CPA‐induced cytotoxicity)
Recommended volumes	Up to 200 ml, common range 0.3–5 ml	Up to 0.5 ml, common range 1–2 μL

### 4.1 Cryopreservation of free organisms

The cryopreservation of living organisms, including cells, bacteria, fungi, and viruses, is a routine procedure in the laboratory.^10^, ^19^ Some of the cryopreserved cells have been widely used in the specific departments of the hospital such as reproductive, blood transfusion, and hematology departments.^20^ For the cryopreservation of living organisms, corresponding protocols can be used as references. Due to the difference in organism composition, the cryobiological responses of different kinds of organisms vary greatly. During the cryopreservation of a particular living organism type, particularly physiological and biological properties should be considered to maximize post‐cryopreservation viability. For example, Lakey et al. summarized the optimal cryopreservation method of varied cells.^21^ In general, for the cryopreservation of pancreatic islets, embryonic stem cells, and oocytes, vitrification is recommended, while slow cooling is suggested for the storage of hepatocytes, hematopoietic stem cells, and mesenchymal stem cells (MSCs).

### 4.2 Cryopreservation of microencapsulation‐based cell delivery systems

Cell microencapsulation is a technology with great promise to treat various diseases.^22^ Biocompatible nature polymers (e.g., alginate, hyaluronic acid [HA], collagen, chitosan, agarose) and synthetic polymer (e.g., gelatin methacryloyl [GelMA], hyaluronic acid methacryloyl, and polyethylene glycol) are used to achieve cell microencapsulation.^23^ Compared with free cells, microencapsulation devices with ideal mechanical properties can provide a supportive environment for cells. Besides, the microencapsulation devices will not influence the permeability of the essential nutrients to cells and can protect the cells from the immune system in some cases.^24^ For the later or emergency use of the cell‐based products, banking is one of the important processes. It is considered that cryopreservation is an excellent way to obtain standardized cell products on demand.

**FIGURE 3:**
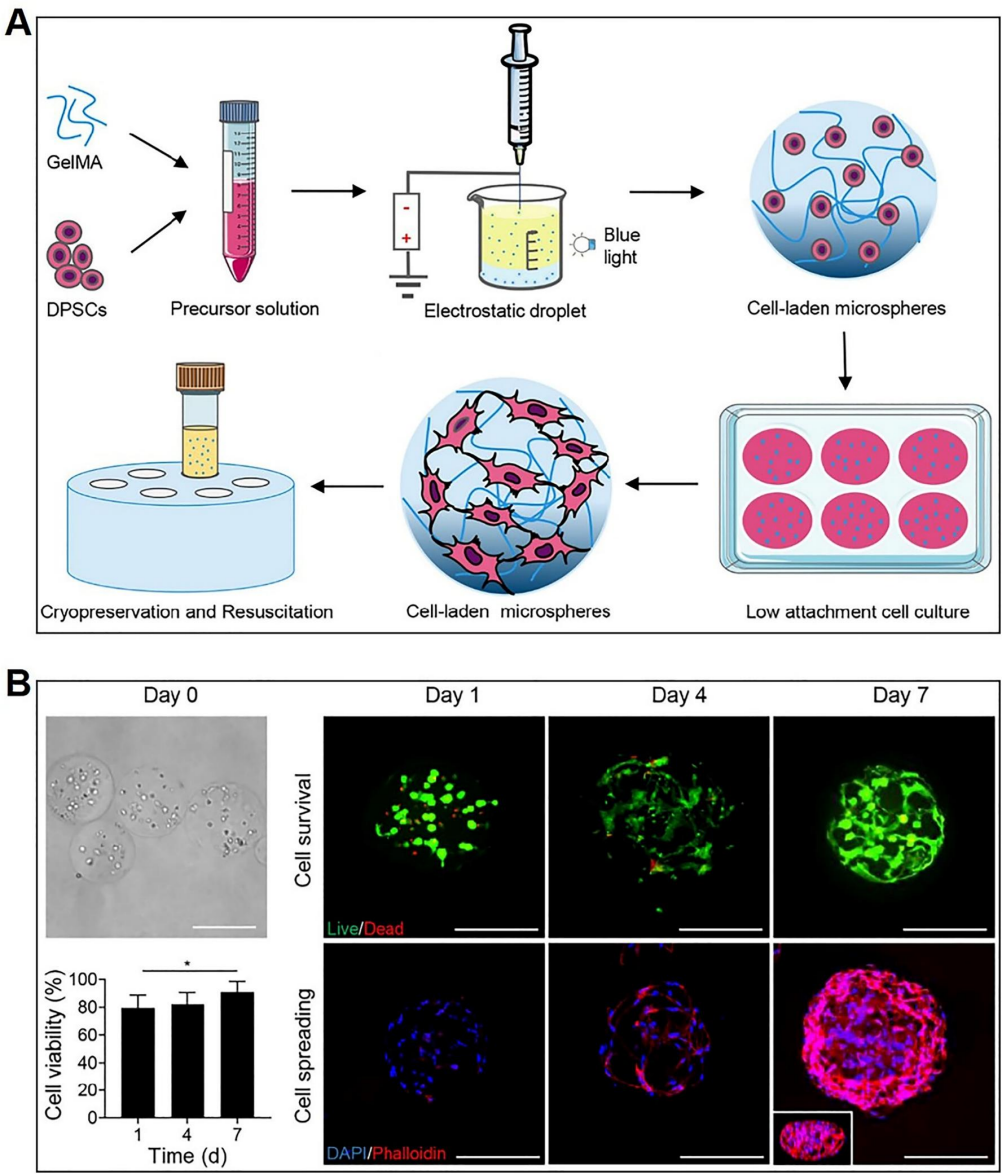
Cryopreservation and resuscitation of cell‐laden microspheres. (A) Schematic illustration of cell‐laden microsphere preparation process. (B) Optical images of the cryopreserved cell‐laden microspheres, and fluorescence images of live/dead staining, cell viability, and F‐actin/nuclei staining of the cryopreserved cell‐laden microspheres after incubation for 1, 4, and 7 days; the insets are the 3D reconstructed images of the microspheres. Scale bars are 200 μm. Reproduced with permission.^29^ Copyright 2021, Elsevier B.V.

Due to the advantages of simple handling and the possibility of large storage volume, slow freezing is often chosen for the cryopreservation of microencapsulated cells.^25^ A variety of alginate‐based microencapsulation has shown higher efficiency in cell cryopreservation compared with traditional free cell cryopreservation.^26^ It was demonstrated that the hydrogel microencapsulation can protect the cells from damage during the freezing process.^27^ Besides, the shape, morphology, and size of the microcapsules have a great influence on the efficiency of the cryopreservation of microencapsulated cells. GelMA is a common photo‐crosslinkable polymer and owns the merits of both natural and synthetic biomaterials, which has attracted considerable attention in tissue engineering.^28^ Recently, Tian et al prepared a GelMA microsphere by electrohydrodynamic spaying method to encapsulate human dental pulp stem cells (hDPSCs) for endodontic regeneration (Figure 3A).^29^ The obtained cell‐laden GelMA microspheres were incubated and then cryopreserved. In brief, 200 μL of microspheres were dispensed in 1 ml of 10% DMSO in a cryovial and then placed in a Mr. Frosty Freezing Container. The whole system was cooled down at 1°C/min, and finally stored at −80°C. After 3 months of cryopreservation, the resuscitated microspheres were cultured in the growth medium for different times. As shown in Figure 3B, the cells in the resuscitated microspheres could proliferate and spread normally.

**FIGURE 4:**
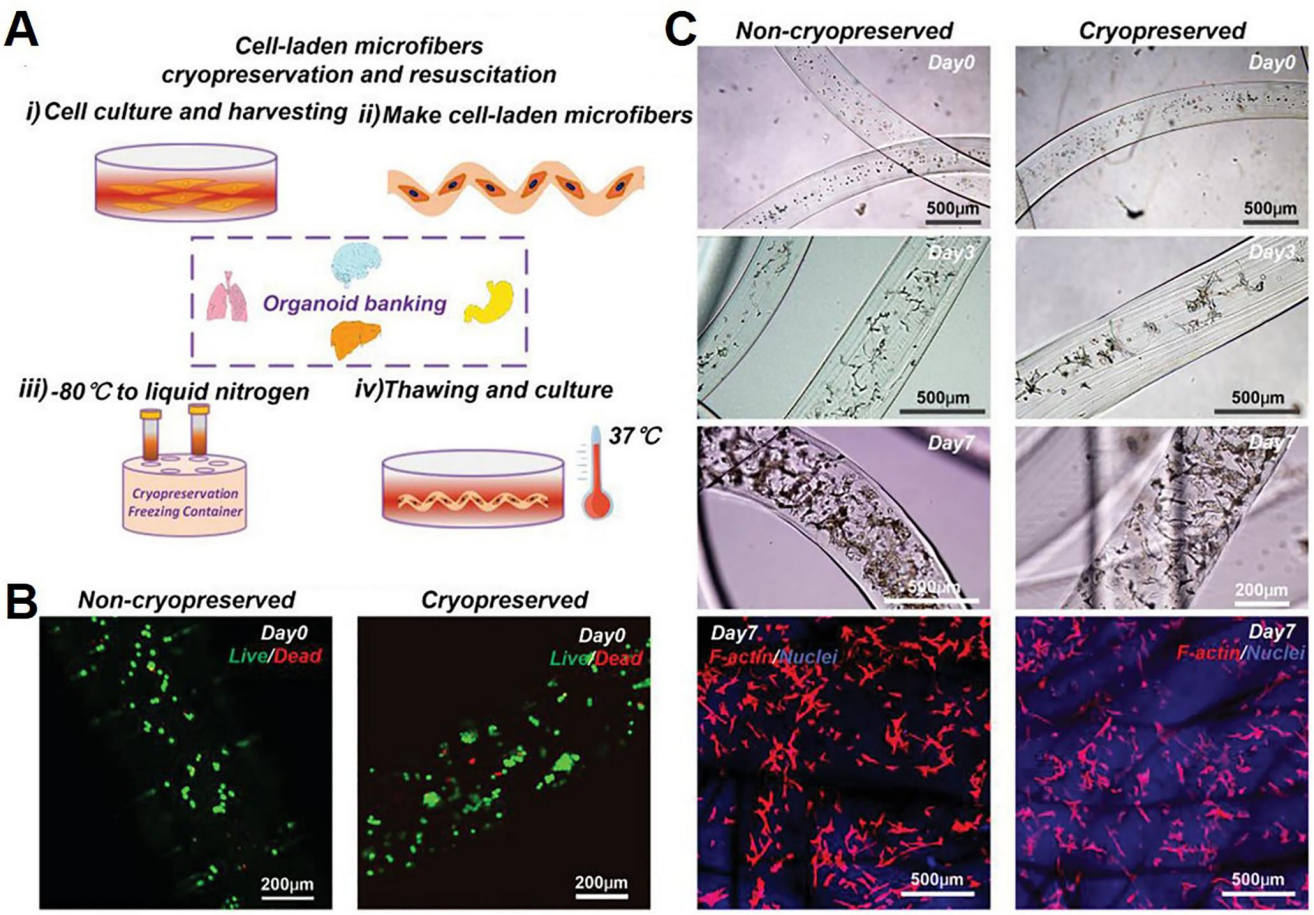
Cryopreservation and resuscitation of cell‐laden microfibers. (A) Schematic illustration of the cryopreservation of cell‐laden microfibers and its potential for organoid banking. (B) Fluorescence images of live/dead staining of non‐cryopreserved and cryopreserved cell‐laden microfibers after 0‐day culture. (C) Optical images of non‐cryopreserved and cryopreserved BMSC‐laden microfibers after 0, 3, and 7‐day culture; and confocal laser‐scanning microscopy images of F‐actin/nuclei staining of non‐cryopreserved and cryopreserved cell‐laden microfibers after 7‐day culture. Reproduced with permission.^31^ Copyright 2019, John Wiley and Sons.

Microfibers are another popular structure for cell microencapsulation. Hydrogel microfibers with excellent biomimetic geometry and bioactivity can provide a unique cell culture platform for diverse applications.^30^ Shao et al. evaluated the cryopreservability of cell‐laden microfibers.^31^ The cryopreservation process of bone marrow‐derived mesenchymal stem cell (BMSC) encapsulated microfibers was displayed in Figure 4A. Following 30 days of cryopreservation, BMSCs in the resuscitated microfibers displayed good viability and growth tendency similar to that in the non‐cryopreserved microfibers (Figure 4B,C). The cryopreservation method greatly improved the availability of cell products based on microencapsulation technology.

### 4.3 Cryopreservation of scaffold‐based cell delivery systems

Scaffolds with a bulky and monolithic nature is another cell delivery system, which consists of films, sponge, electrospun nanofibers, etc.^3^ ^a^ Compared with microencapsulated cells, which are much closer to free cells, the cryopreservation of the cell delivery scaffolds faces more obstacles, including non‐uniform distribution of CPAs for cells adherent or located deep in the bulk, compromised accuracy of temperature control, and uneven shrinkage or expansion of the matrices, etc.^17^ Although with much difficulty, many research studies have demonstrated the possibility of the long‐term cryogenic storage of scaffold‐based cell delivery systems Multiple parameters, such as cell types, properties of the scaffold materials, cell culture conditions before freezing, and CPAs, should be taken into consideration for the successful cryopreservation.^32^

**FIGURE 5:**
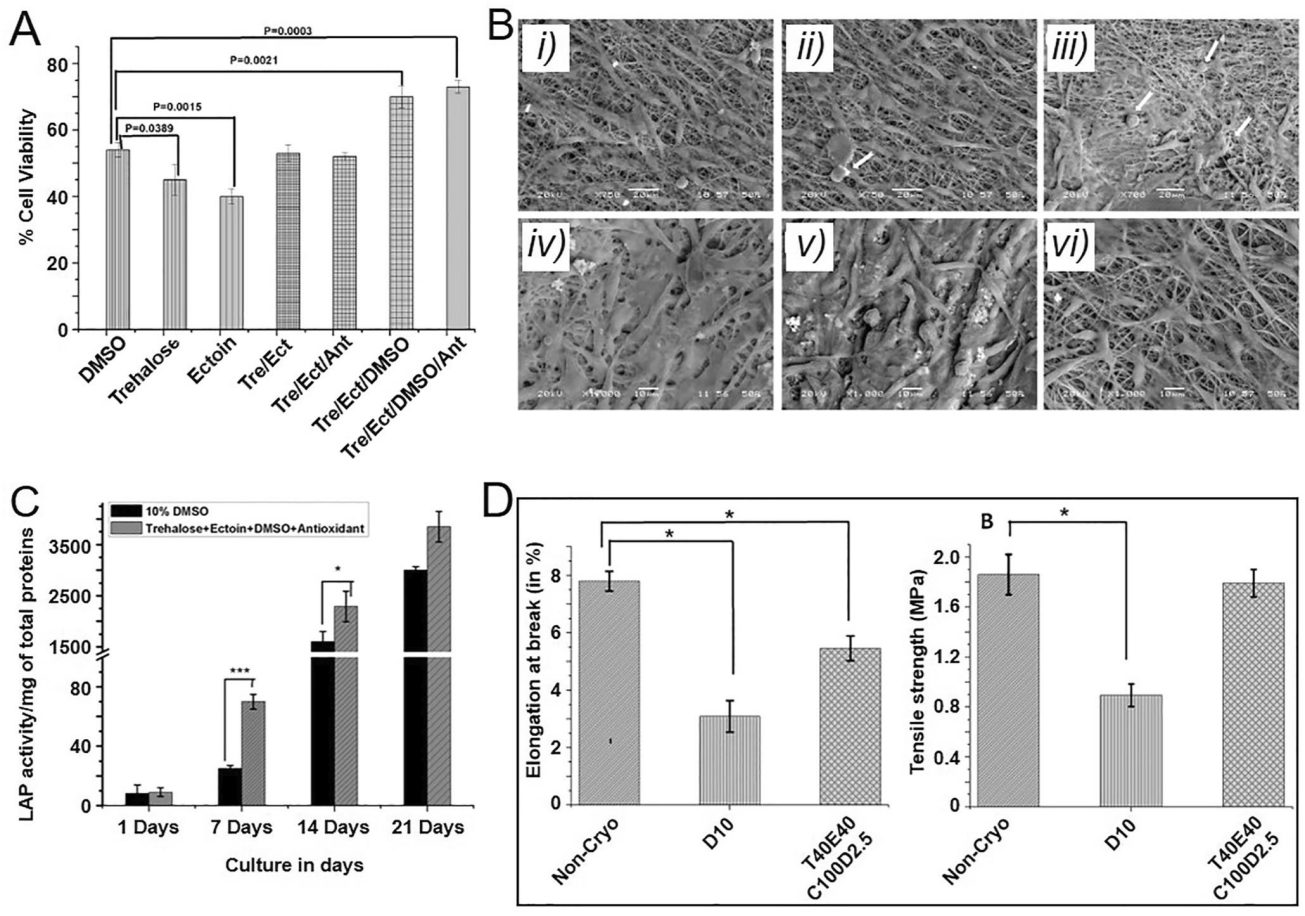
Characterization of cryopreserved electrospun nanofibers seeded with cells. (A) Cell viability after 24 h of cryopreservation. (B) Scanning electron microscopy (SEM) images of cryopreserved nanofibers with different CPAs. Non‐cryopreserved nanofibers for 1 day (i) and 14 days (iv). Cryopreserved nanofibers with T40/E40/A100/D2.5 for 1 day (ii) and 14 days (v). Cryopreserved nanofibers with DMSO for 1 day (iii) and 14 days (vi). Arrows indicate the improper attachment of cells with rounded morphology. (C) ALP activity on day 1, 7, and 21 of post‐thaw cells cultured on the nanofibers. (D) Elongation at breakage and tensile strengths of the nanofibers. Reproduced with permission.^33^ ^b^ Copyright 2014, Elsevier Inc.

Due to the larger sample volumes of the scaffold‐based cell delivery systems, slow freezing is preferable.^33^ Costa et al. fabricated a porous fiber mesh with poly(caprolactone) and starch.^33^ ^a^ The porous fiber meshes were seeded with goat BMSCs and cryopreserved with 10% DMSO in liquid nitrogen. The cell viability and fiber mesh properties after 7 days of cryopreservation were maintained well. Compared with nonporous discs, the fiber meshes displayed better cell retention and cell viability during the cryopreservation process because of the porosity and interconnectivity of the matrices. Pramanik et al. used silk fibroin electrospun nanofibers seeded umbilical cord‐derived MSCs to compare different CPA formulations.^33^ ^b^ Results showed that T40/E40/A100/D2.5 with a mixture of 40 mM trehalose, 40 mM ectoin, 100 μg catalase, and 2.5% DMSO was most effective. The viability, proliferation, and functions of cells cryopreserved with T40/E40/A100/D2.5 were superior to that of cells cryopreserved with 10% DMSO (Figure 5A–C). In addition, the mechanical properties of the cryopreserved electrospun nanofibers were unchanged (Figure 5D). A porous scaffold made by polyvinyl formal resin, which was immobilized with NIH/3T3 fibroblasts, was used to study the influence of collagen coating and preculture on the performance of scaffold cryopreservation.^33^ ^c^ The results demonstrated that the collagen coating could enhance immobilization efficiency when cell seeding, and 1 day preculture before the cryopreservation significantly improved the cell growth after resuscitation.

**FIGURE 6:**
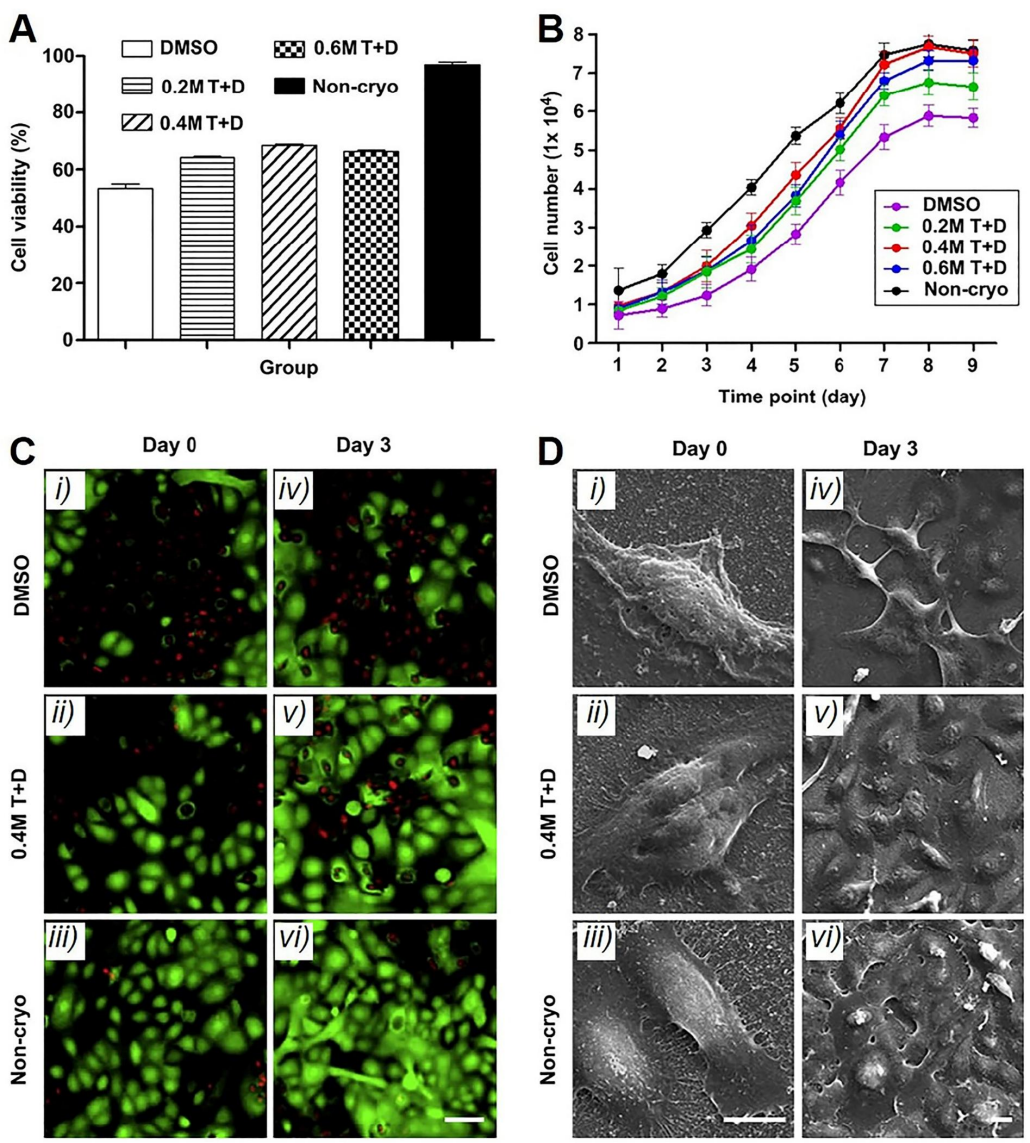
Characterization of cryopreserved chitosan‐gelatin membranes seeded with cells. (A) Cell viability after 1‐month cryopreservation. (B) Cell numbers at each time point from various groups. (C) Fluorescence images of live/dead staining and (D) SEM images of keratinocytes seeded on the membranes immediately after and 3 days following cryopreservation. Scale bars are 50 μm (C), 10 μm (D i, ii, iii), and 10 μm (D iv, v, vi), respectively. Reproduced with permission.^34^ ^a^ Copyright 2011, Elsevier Ltd.

There are several studies about the cryopreservation of scaffold‐based cell delivery systems via vitrification.^34^ Cao et al. transferred the prepared chitosan‐gelatin membranes seeded with human keratinocytes to cryovials containing the optimal cryopreservation solution (0.4 M trehalose and 10% MDSO).^34^ ^a^ After being kept at 4°C for 30 min, the membranes were placed in liquid nitrogen for 1 month. The addition of trehalose enhanced cell viability and proliferation (Figure 6A–D). Besides, a polycaprolactone‐gelatin nanofibrous scaffold loaded with porcine BMSCs was also stored in liquid nitrogen, during which 40% EG and 0.6 M sucrose were as CPAs.^34^ ^b^ Brockbank et al. used a perfusion concentric cylinder bioreactor to cryopreserve a poly‐L‐lactide porous scaffold with bovine chondrocytes.^34^ ^c^ The CPA formulation of DMSO (3.97 M), formamide (3.97 M), and 1,2‐propanediol (2.83 M) resulted in the best post‐vitrification cell viability levels.

**FIGURE 7:**
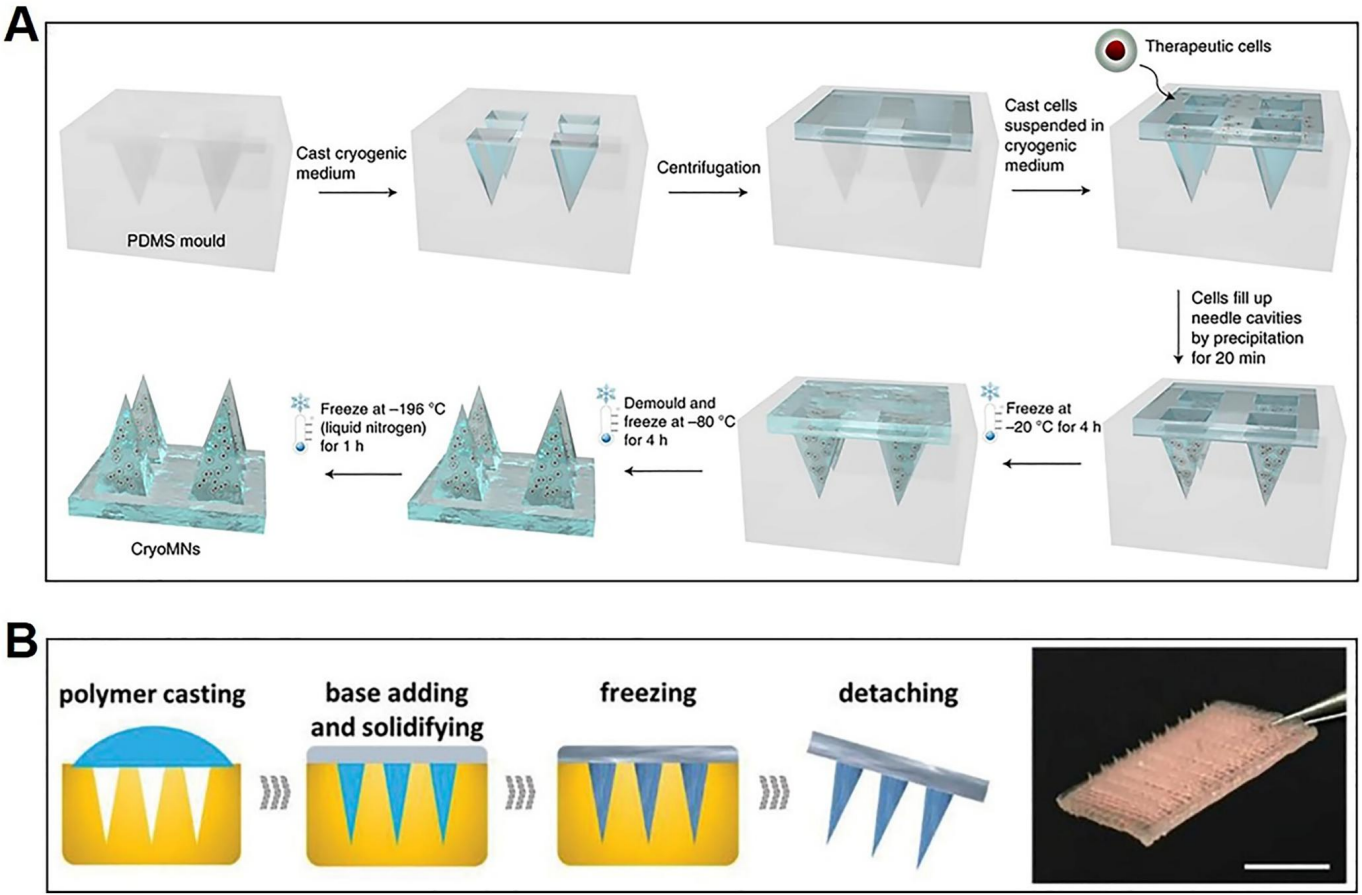
The preparation of cryoMNs and ice MNs. (A) Schematic of cryoMN fabrication. Reproduced with permission.^36^ Copyright 2021, Springer Nature. (B) Schematic of ice MN preparation process and photograph of an ice MN patch. Scale bars are 0.5 cm. Reproduced under terms of the CC‐BY license.^38^ Copyright 2021, The Authors, published by John Wiley and Sons.

Different from the following cryopreservation after the preparation of the scaffold‐based cell delivery systems, it is a novel idea to add the freezing strategies of cryopreservation into the scaffold production process. By integrating with living organisms, microneedles with the advantage of minimal invasion can perform a variety of functions.^35^ Inspired by the conventional cryopreservation technology, a novel MN was prepared by Xu et al.^36^ As shown in Figure 7A, the cryomicroneedles (cryoMNs) were fabricated by freezing stepwise in a microneedle template. In this process, DMSO (2.5%) and sucrose (100 mM) were as CPAs. After being transferred from the storage condition, the cryoMNs could be easily inserted into the porcine skin within 40 s. After melting in PBS at 37°C, cell viability of six types of cells loaded in the cryoMNs varied from 20%–55%, and all the cells display good proliferation ability. Besides, melanocytes and RFP‐HeLa cells in cryoMNs displayed minimal viability change after 1 month of preservation. In another work of this team, the process was further simplified.^37^ The buffer solutions composed of predatory bacteria, glycerol, and PBS were added to the MN mold and then centrifuged to fill up the needle cavities. After some other solutions were added as the base, they were placed at 4°C for the precipitation of bacteria. After storage at −20°C and at −80°C for 4 h orderly, the MNs were peeled off from the molds and the cryoMNs were obtained.

Apart from cryoMNs, Zhao et al. also present another MN by freezing method.^38^ After the conventional template replication method, a freezing step was introduced to prepare ice MNs. In brief, the precursor solutions were cast into the cavities of the MN template and then polymerized. After the covering of soggy gauze, the whole system was frozen and then detached from the template to obtain ice MNs (Figure 7B). In theory, the ice microneedles could be made from almost all water‐containing materials (e.g., water, GelMA, alginate, Matrigel). After the freezing step, these soft materials can be converted into hardness to achieve satisfactory penetration into the skin. Because all preparation procedures were mild, the ice MNs could load varied components from small molecules, and macromolecules to even living organisms without losing their activity.

## 5 BIOMEDICAL APPLICATIONS OF FREEZING BIOLOGICAL ORGANISMS

Biological organism‐based therapy is a promising direction of biomedicine. By the freezing method, living organisms or inactivated organisms can be easily obtained. Engineered inactivated cancer cells by the freezing strategies of cryoablation have attracted great attention in cancer immunotherapy. Cryopreservation can achieve pooling and long‐term storage of the cells that would be utilized for transplantation (including pancreatic islet, hepatocyte, bone marrow, etc.), blood transfusion, artificial insemination, and in vitro fertilization, and off‐the‐shelf availability of these cells is feasible.^20^, ^39^ The cryopreserved microencapsulation‐ and scaffold‐based cell delivery systems have potential in transplantation and tissue regeneration. cryoMNs and ice MNs via the freezing method have been used in living organism‐based therapy. Altogether, freezing biological organisms has a wide range of applications in biomedicine. In this section, some important biomedical applications of freezing biological organisms, including transplantation, tissue regeneration, anti‐infection therapy, and anti‐tumor therapy, will be introduced in detail.

### 5.1 Transplantation

Pancreatic islet transplantation is an excellent alternative as a type I diabetes treatment, in addition to drugs.^40^ After introducing the Edmonton protocol in 2000, islet transplantation procedures and immunosuppression regimes have been optimized, which has greatly improved the efficacy of islet transplantation.^41^ It is reported that about 70% of patients can remain insulin independent 1 year after transplantation, and the 5‐year survival rate of the grafts is 82%.^42^ However, the insufficient islets available for transplantation remains a challenge. Cryopreservation allows the long‐term storage of islets from multiple donors and solves the problem of availability for donor islet transplantation.^43^ According to the method reported by Davis et al., the islets were placed in a cryotube with pre‐added CPAs, equilibrated at 4°C for 25 min, and then placed in a freezer. It was firstly lowered from 4°C to −9°C at a rate of 2°C/min, to −40°C at a rate of 0.3°C/min, and to −140°C at a rate of 10°C/min, and finally directly put into liquid nitrogen for storage. This method has been proven to be effective in reducing cell metabolism so that cells can be stored for a long time, and play a role in protecting cells.^44^

**FIGURE 8:**
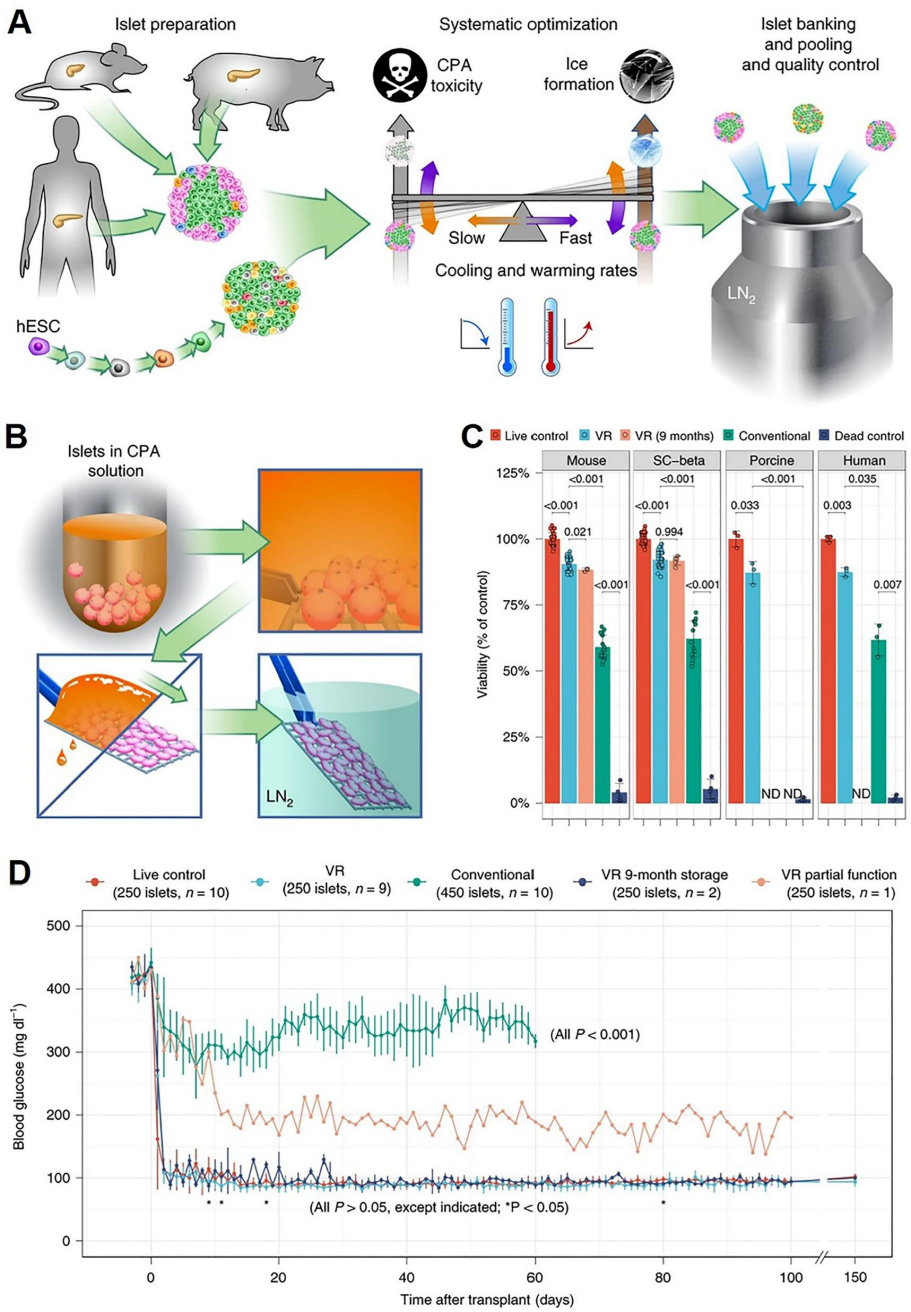
Novel vitrification and rewarming (VR) cryopreservation of pancreatic islet using cryomesh. (A) Schematic of the systematic investigation. hESC, a human embryonic stem cell. (B) Schematic of the use of cryomesh. (C) Viability of mouse, SC‐beta, porcine, and human islets from treatment groups including live control, VR, VR 9 months (islets stored in liquid nitrogen for 9 months before rewarming), conventional and dead control (treated by 75% ethanol). ND, not done. (D) Blood glucose levels of streptozotocin‐induced diabetic mice after syngeneic transplant of marginal mass mouse islets (250 islets per recipient) from treatment groups, including live control, VR, VR 9 months, and conventional cryopreservation (450 islets per recipient). Reproduced under terms of the CC‐BY license.^47^ Copyright 2022, The Authors, published by Springer Nature.

Vitrification is increasingly used for islet cryopreservation.^45^ Despite numerous satisfactory results, one drawback of this method is the use of high‐concentration CPAs, which are toxic to cells.^46^ Minimizing the exposure to CPAs will complicate the handling process. Recently, Finger et al. performed a new cryopreservation method for pancreatic islet transplantation by systematically investigating CPAs and the vitrification method (Figure 8A).^47^ The optimal CPA formulation of 22% EG and 22% DMSO was used. By using cryomesh, excess CPA was removed (Figure 8B). After vitrification and rewarming (VR), the islets showed high cell survival even after 9 months of cryogenic storage (Figure 8C). In mice models, 92% of the recipients were cured of diabetes within 24–48 h after the transplantation of these cryopreserved islet cells, and excellent glycemic control was achieved for 150 days (Figure 8D). The study suggested that this novel cryopreservation regime is a powerful method to improve the islet supply, allowing the pooling of islets from multiple pancreases to improve transplant outcomes for the treatment of diabetes.

**FIGURE 9:**
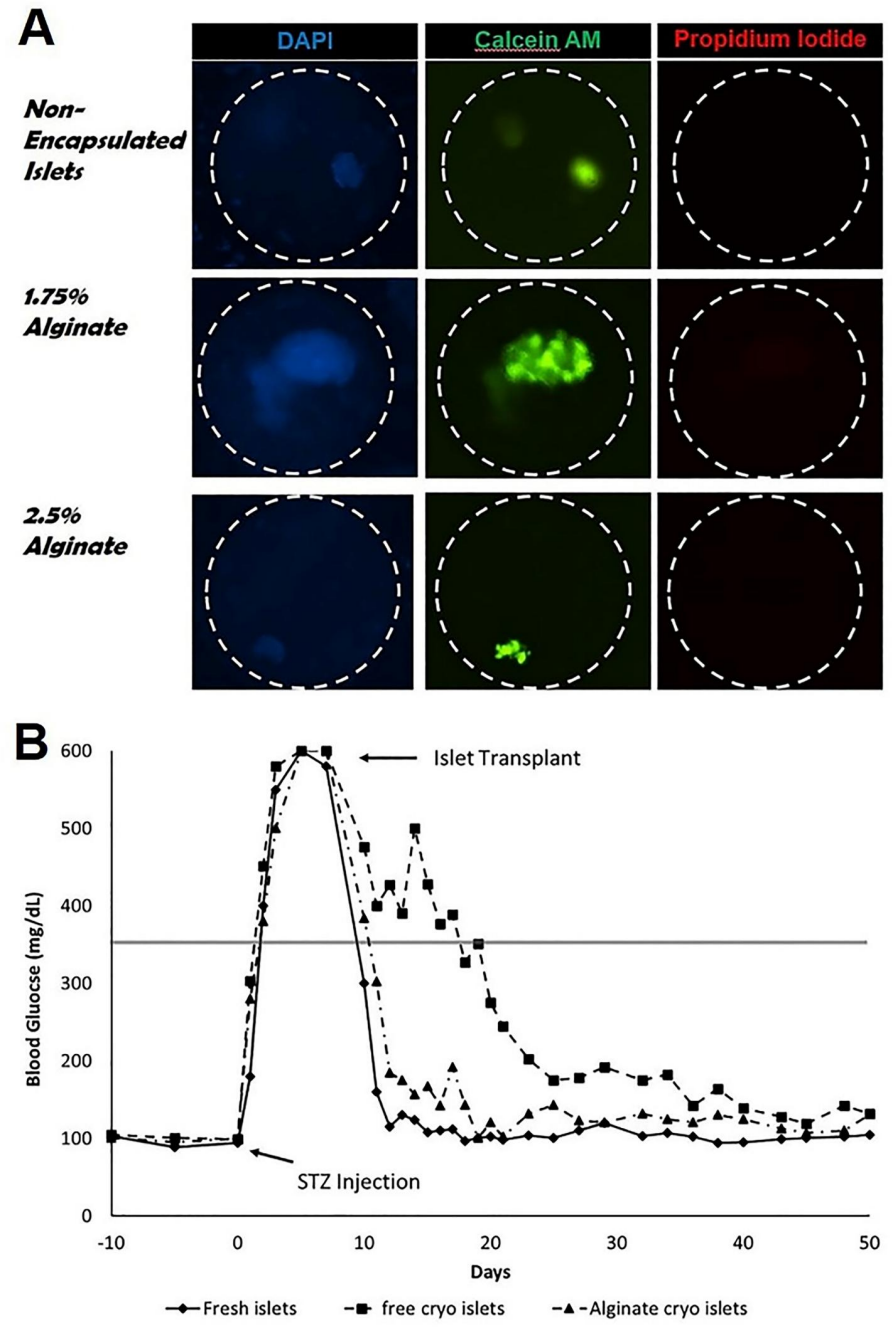
Cryopreserved alginate‐encapsulated islets for the treatment of diabetes. (A) Images of islet viability after 1‐month cryopreservation. (B) Blood glucose profiles of streptozotocin‐induced diabetic nude mice transplanted with fresh islets, cryopreserved islets, and cryopreserved encapsulated islets. On day 7 after streptozotocin injection, the arrow indicates the 1000 islets transplanted intraperitoneally (for encapsulated islets due to size) or in the kidney capsule (for free islets). Reproduced with permission.^50^ Copyright 2019, The Authors, published by SAGE Publications.

In addition to optimizing CPA formulation and freezing/thawing rate, there are some other methods to improve pancreatic islet cryopreservation outcomes.^44^, ^48^ As previously mentioned, microencapsulation of cells before cryopreserving is a promising approach for future transplantation. Inaba et al. first demonstrated the merits of alginate‐based microencapsulation in pancreatic islets cryopreservation.^49^ Lakey et al. compared the cryopreservation outcomes of alginate with different concentrations and found that 1.75% of alginate microencapsulation owned higher survival after the cryogenic storage (Figure 9A).^50^ In the transplant experiment, the time of reaching normoglycemia after transplantation was 5 days for the mice treated with 1.75% alginate‐encapsulated cryopreserved islets, a little higher than the mice treated with fresh islets (4 days), while significantly lower than the mice treated with unencapsulated cryopreserved islets (18 days) (Figure 9B). In another study, normoglycemia of the rats with cryopreserved microencapsulated islet transplantation could maintain for 460 days.^51^

Except pancreatic islet transplantation, other cryopreserved cell transplantations have been explored. Hepatocyte transplantation is a new type of treatment for multiple types of liver disease such as liver failure and metabolic diseases.^52^ During the process of hematopoietic stem cell transplantation, normal hematopoietic stem cells from donors are injected intravenously into patients with blood disorders such as leukemia, severe aplastic anemia, and refractory recurrent lymphoma.^53^ In general, cryopreservation protocols for hepatocyte and hematopoietic stem cells follow the traditional slow cooling method.^54^ Similarly, the microencapsulated hepatocytes have been used to transplant and showed the potential for in vivo treatment of liver diseases.^26^ ^c,^ ^55^ In summary, successful cryopreservation is essential for cell transplantation. On the one hand, sufficient cells can be accumulated for effective transplantation. On the other hand, the banking of these cell products can provide timely treatment for patients on demand.

### 5.2 Tissue regeneration

Recent years have witnessed great progress in tissue regeneration. Biomaterials with native or modified living cells are widely used to build tissue‐engineered constructs as the equivalents of the skin, bone, cartilage, muscle, and other tissues.^56^ The storage of cell‐biomaterial composited products is essential for tissue engineering.^57^ Kofron et al. prepared the thin films or microspheres from poly(lactide‐co‐glycolide) to adhere SaOS‐2 cells.^58^ After cryopreservation by slow freezing method, higher cell survival was observed compared with the same suspension cells. Cao et al fabricated a tissue engineered bone consisting of a partially demineralized bone matrix and osteo‐induced canine BMSCs.^34^ ^d^ After 3 months of cryopreservation by vitrification with a novel vitreous solution of 40% DMSO, 40% Euro Collins, and 20% basic culture medium, their survival, and osteogenic potential maintain well. The feasibility of cryopreservation of scaffold‐based chondrocyte and myoblast delivery systems was also verified.^34^ ^c,^ ^59^

**FIGURE 10:**
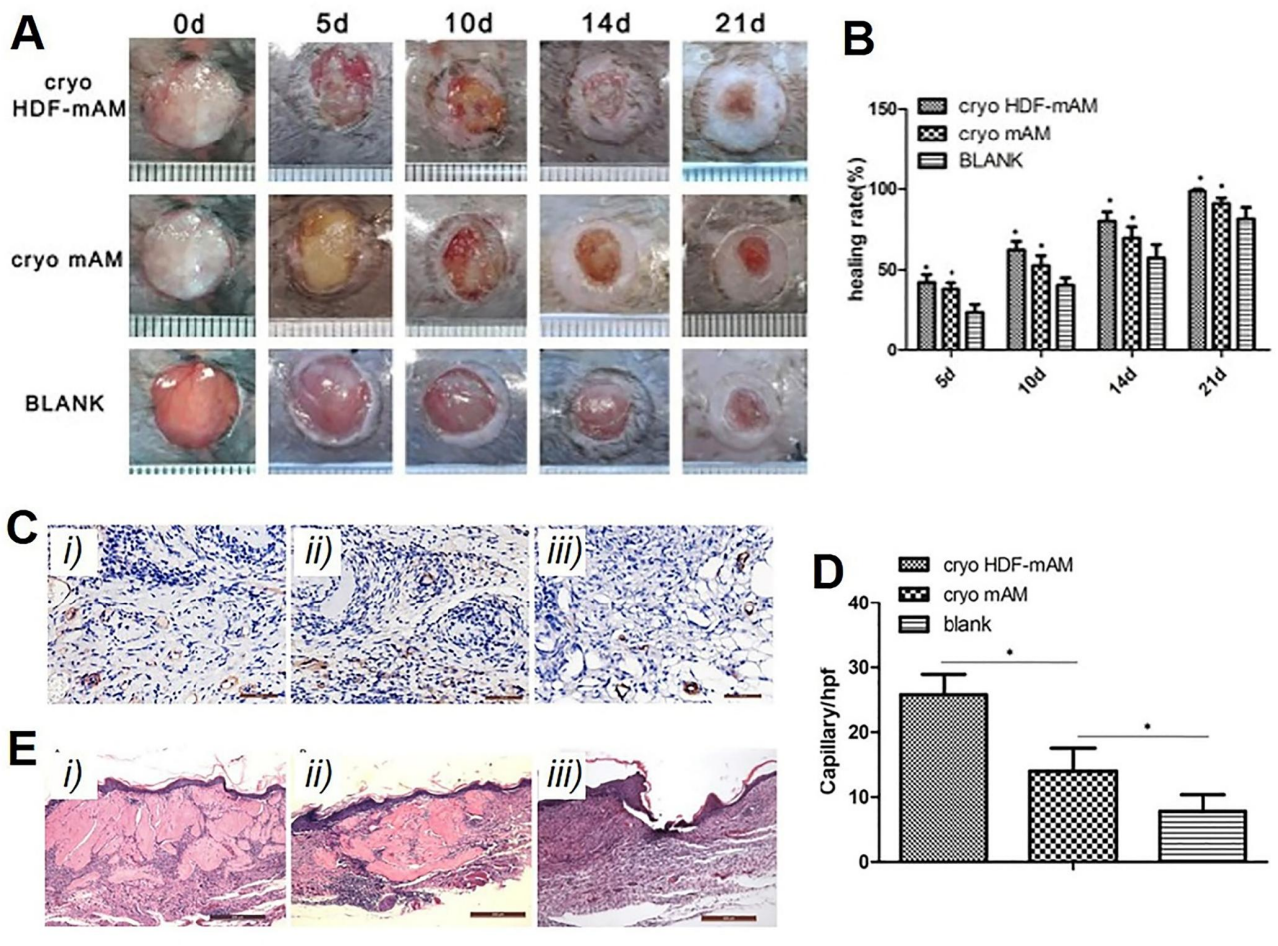
Cryopreserved HDF‐mAM for wound healing of full‐thickness skin defects in db/db mice. (A) Photographs of wounds with different treatments. (B) Quantitative analysis of wound healing rate. (C) Specimens from day 14 wounds stained with CD31 in cryopreserved HDF‐mAM group (i), cryopreserved mAM group (ii), and blank group (iii). Scale bars are 100 μm. (D) Quantitative analysis of neovascularization. (E) HE staining of sections at day 28 after transplantation in cryopreserved HDF‐mAM group (i), cryopreserved mAM group (ii), and blank group (iii). Scale bars are 100 μm. Reproduced with permission.^61^ Copyright 2015, e‐Century Publishing Corporation.

Tissue‐engineered skins are useful in wound healing, and optimized storage of them needs to be developed. Kuroyanagi et al. created an allogeneic dermal substitute composed of human dermal fibroblasts and a two‐layered sponge of HA and atelocollagen.^60^ The dermal substitute could be stored at −152°C and −85°C for 1 year and 6 months, respectively, with sufficient cell viability, proliferation ability, and vascular endothelial growth factor (VEGF) release. Zheng et al. used a 3D micronized amniotic membrane (mAM) in combination with a rotary cell culture system to culture and amplify human dermal fibroblasts (HDF) for the construction of a dermal substitute HDF‐mAM.^61^ After 6 months of cryopreservation, HDF‐mAM was transplanted to the full‐thickness skin defects of db/db mice of the diabetes model. As shown in Figure 10A,B, cryopreserved HDF‐mAM promoted wound healing significantly. Besides, neovascularization was significantly promoted and the wounds were completely healed, indicating the acceleration of diabetic wound healing (Figure 10C–E).

### 5.3 Anti‐infection therapy

**FIGURE 11:**
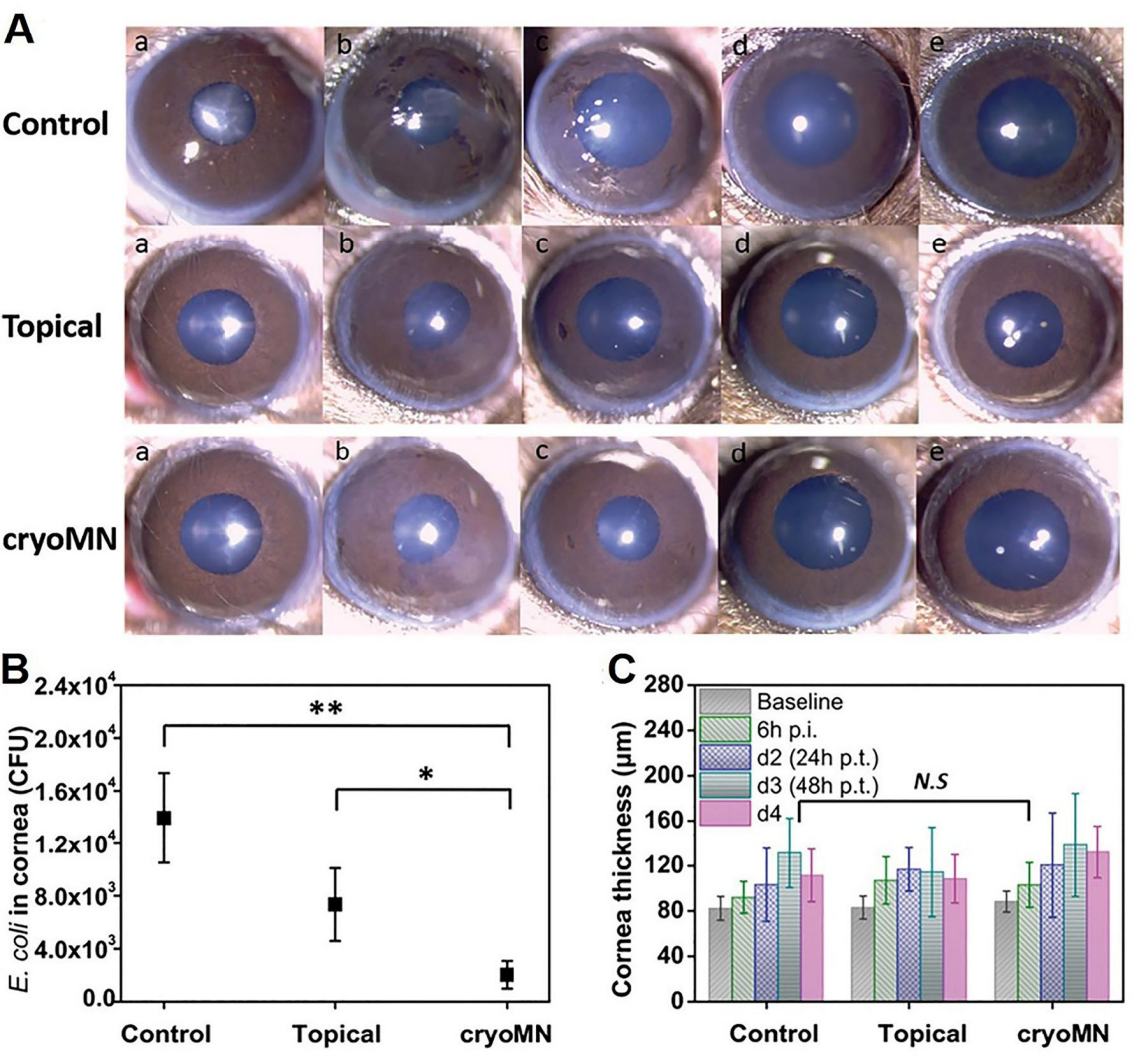
Predatory bacteria‐loaded cryoMNs for eye infection. (A) Cornea images were taken by slit‐lamp photography. (a) Baseline; (b) 6 h p.i. after inoculation (prior treatment); (c) Day 2 (24 h post‐treatment [p.t.]); (d) Day 3; (e) Day 4. (B) Final *E. coli* concentration inside mouse corneas. (C) Cornea thickness before and after treatment every day. Reproduced under terms of the CC‐BY license.^37^ Copyright 2021, The Authors, published by John Wiley and Sons.

Infection is a common disease, and some severe infections can be life‐threatening. Conventionally, antibiotics are the common treatment method. However, antibiotic abuse has caused the emergence of drug‐resistant bacteria.^62^ As an alternative approach, predatory bacteria is used to eliminate antimicrobial‐resistant bacteria.^63^ To achieve the deep penetration of the predatory bacteria, cryoMNs were used as a topical delivery platform to deliver *Bdellovibrio bacteriovorus* (*B. bacteriovorus*) for the treatment of eye infection (Figure 11A).^37^ Bacterial viability in cryoMNs could remain above 80% after the long‐term cryo‐storage. The predatory bacteria encapsulated cryoMNs displayed excellent inhibition effect on gram‐negative bacteria. In a rodent eye infection model, the infection was decreased by nearly six times in the cryoMN treatment group without no obvious change in the cornea thickness and morphology (Figure 11B,C).

**FIGURE 12:**
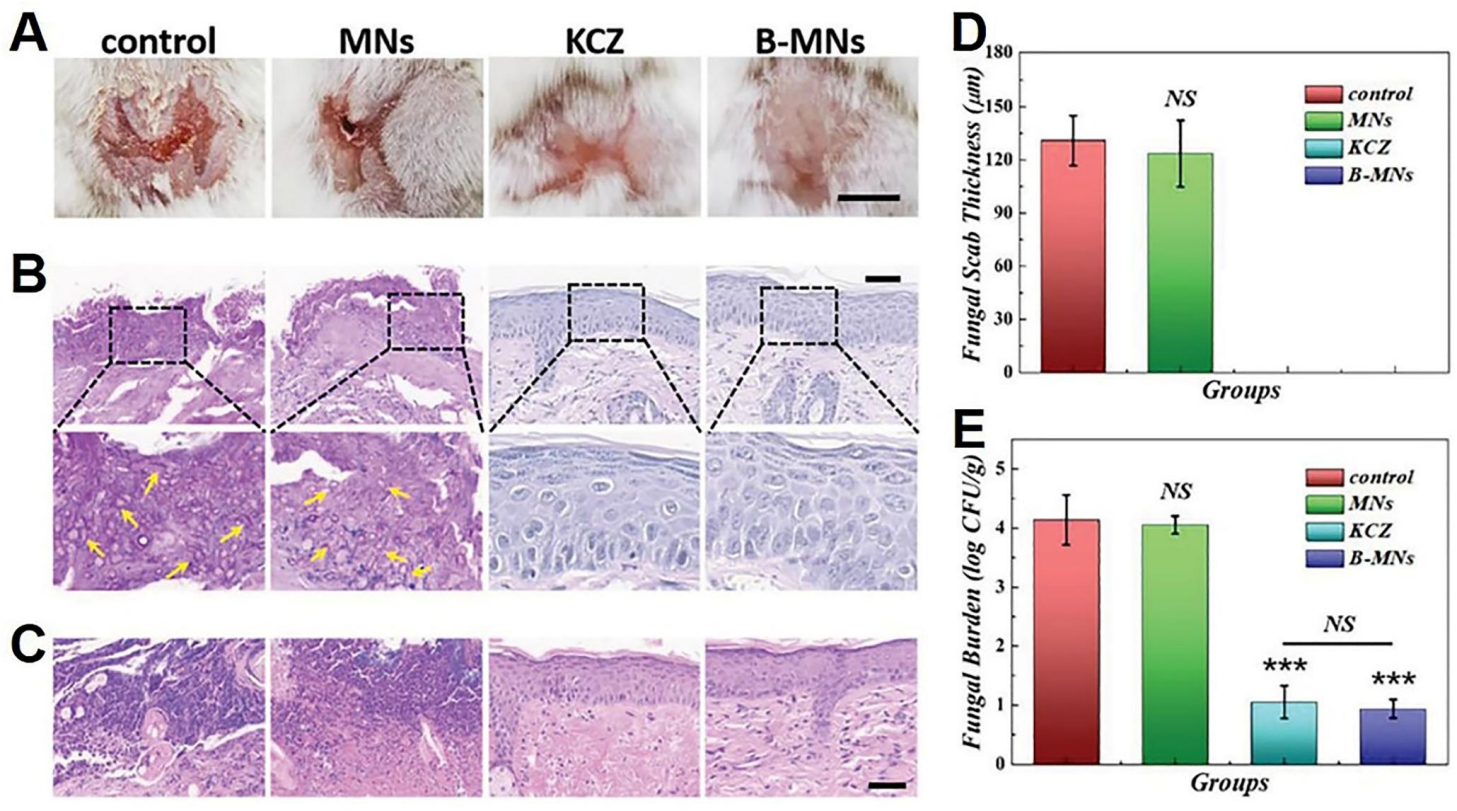
*B. subtilis*‐loaded ice MNs for skin fungal infection. (A) Representative photos of back skins of the mice in the control group, the MNs group (treated with MNs), the KCZ group (treated with ketoconazole cream), and the B‐MNs group (treated with *B. subtilis*‐loaded ice MNs) on day 13. Scale bars are 0.5 cm. (B) PAS staining of mouse back skins in different groups on day 13. The arrows pointed to the pseudohyphae of *C. Albicans*. Scale bars are 50 μm. (C) H&E staining of mouse back skins in different groups on day 13. Scale bars are 50 μm. (D) Quantitative analysis of the fungal scab thicknesses of the mice. (E) Quantitative analysis of fungal burden of the infected skin tissues. Reproduced under terms of the CC‐BY license.^38^ Copyright 2021, The Authors, published by John Wiley and Sons.

In addition to bacterial infections, fungal infections can be treated by predatory bacteria. One of the typical applications of ice MNs is to deliver beneficial bacteria for the treatment of fungal infections.^38^ After freezing *Bacillus subtilis* (*B. subtilis*)‐encapsulated GelMA MNs, ice MNs (B‐MNs) were obtained and used in the mice with skin fungal infection (*C. Albicans*). After 12 days, no serious infection was observed in the mice of the KCZ group (ketoconazole cream) and the B‐MNs group (Figure 12A). Almost no pseudohyphae and only a few inflammatory cells could be seen in these two groups (Figure 12B,C). Besides, both fungal scab and fungal burden were significantly low in the B‐MNs group, which was comparable to commercial drugs (Figure 12D,E). These results indicated the satisfactory effects of antifungal and skin recovery of B‐MNs.

### 5.4 Anti‐tumor therapy

**FIGURE 13:**
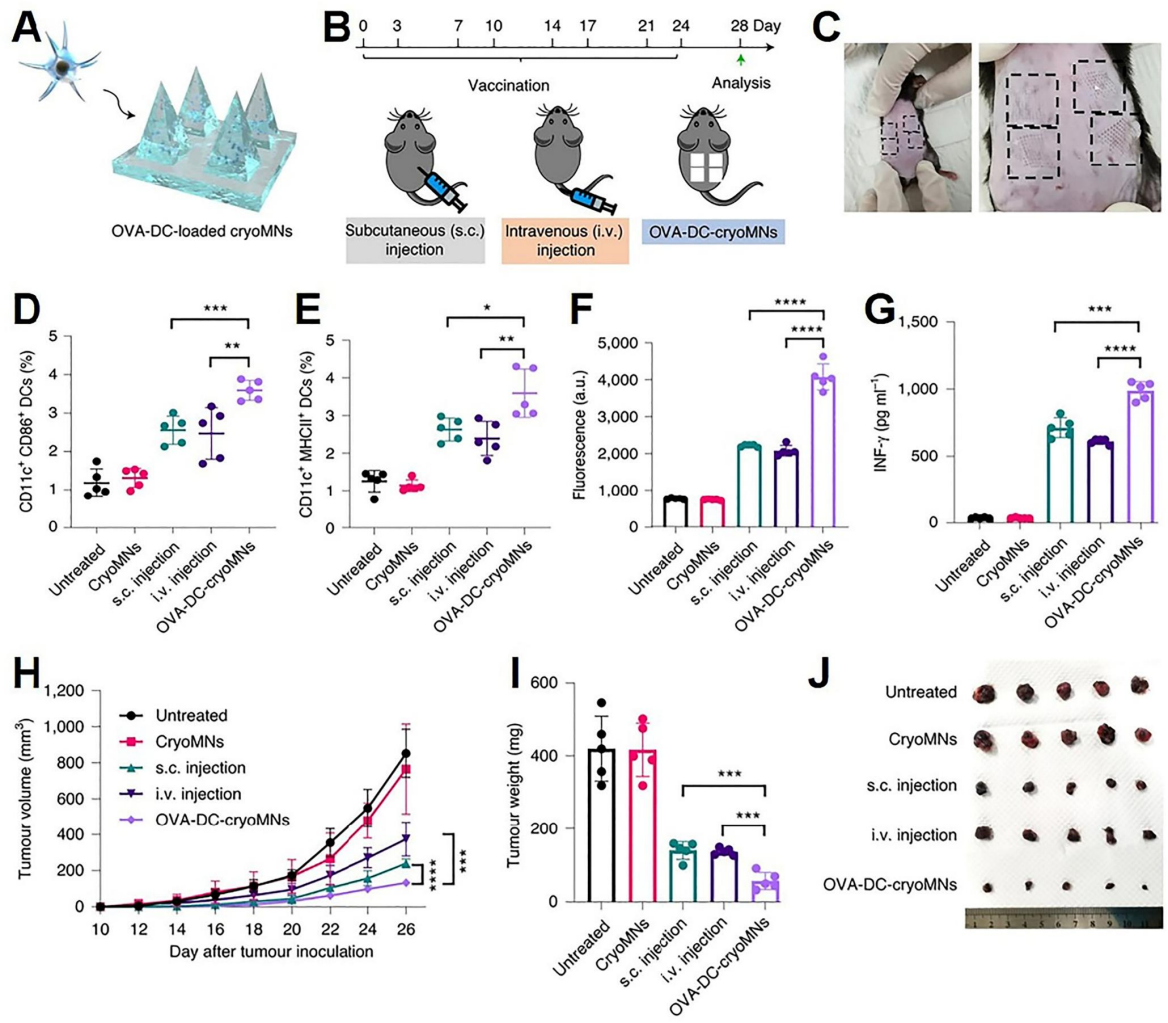
OVA‐DC‐loaded cyoMNs for cancer treatment. (A) Schematic of loading OVA‐DCs in cryoMNs. (B) The protocol of vaccination with OVA‐DC‐cryoMNs in mice. (C) Photograph of the administration sites of OVA‐DC‐cryoMNs on the back skin of mice (left) and an enlarged photograph of the same mouse (right). The sites are bounded by black dashed lines. Quantification of the percentage of CD11c^+^CD86^+^ DCs (D) and CD11c^+^ MHCII^+^ DCs (E) in draining lymph nodes excised from mice in different treatment groups. (F) In vitro proliferation of extracted splenocytes restimulated with 50 μg ml^−1^ antigen (OVA). (G) Secretion level of IFN‐γ in the culture supernatants after 48 h of culture. (H) Average tumor growth of mice bearing established B16‐OVA melanoma tumors after different vaccination treatments. (I) Weight and (J) photographs of excised tumors on day 26. Reproduced with permission.^36^ Copyright 2021, The Authors, published by Springer Nature.

Immunotherapy plays an increasingly important role in cancer treatment. Tumor immunotherapy includes CART, cancer vaccines (including DC vaccines, whole‐cell tumor vaccines, etc.), immune checkpoint inhibitors, etc.^64^ Among them, cell‐based immunotherapy including CART and DC are delivered via bolus injection, which leads to limited cytotoxic T cell infiltration in the tumor or inefficient homing in the lymphatic organs.^65^ Due to the ability of local delivery and keeping the cells active, cryoMNs are a suitable means to solve this problem. cryoMNs were used to load ovalbumin (OVA)‐pulsed DC (OVA‐DC) to construct the DC vaccine (Figure 13A).^36^ After vaccination with four patches twice per week, the immune cells from lymph nodes and spleen were harvested to analyze (Figure 13B,C). Higher mature DC percentage in lymph nodes, faster proliferation, and higher IFN‐γ levels of splenocytes were observed in mice vaccinated with OVA‐DC‐cryoMNs than those of mice vaccinated with conventional subcutaneous and intravenous injection of OVA‐DCs (Figure 13D–G). In mice inoculated with B16‐OVA cells, OVA‐DC‐cryoMNs displayed slowest tumor growth (Figure 13H–J). The cryoMNs provide a novel direction for a range of cell‐based immunotherapy such as DC vaccine and CART.

**FIGURE 14:**
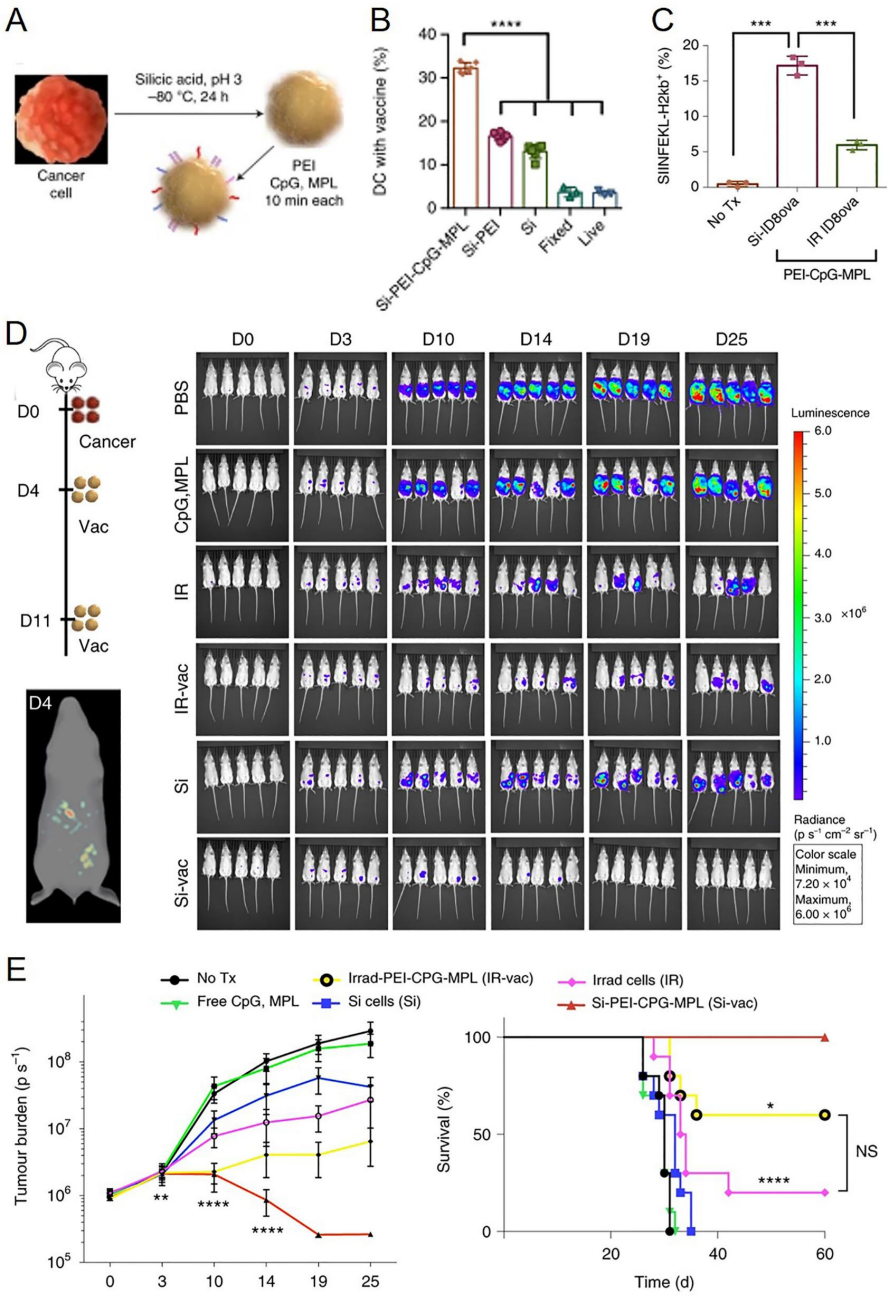
Cancer vaccines from cryogenically silicified tumor cells functionalized with pathogen‐associated molecular patterns (PAMPs) for tumor treatment. (A) Cryo‐silicification and adsorption of PAMPs to cancer cells. Blue motifs, CpG; purple motifs, MPL; red motifs, PEI. (B) Flow cytometry analysis of DC uptake of Si BR5‐Akt cells presenting no TLR ligands (Si), PEI, or PEI‐CpG‐MPL. (C) Flow cytometry analysis of MHC I presentation of tumor antigen (SIINFEKL‐H2Kb) on DC 72 h after the addition of ID8ova vaccine cells or control irradiated ID8ova cells. (D) Diagram of the treatment schedule and tumor burden (IVIS 2D and 3D images) in FVB mice IP injected with BR5‐Akt‐Luc2 tumor cells, followed by treatment with free adjuvant (CpG, MPL) or vaccination with Si or irradiated (IR) cancer cells, with and without adjuvant. (E) Tumor burden and survival curves of mice with different treatments. Reproduced with permission.^15^ Copyright 2021, The Authors, published by Springer Nature.

Similar to other whole cell cancer vaccines, inactivated cancer cells by freezing method still face the challenge of the limited anti‐tumor immune response. One solution is to enhance immunogenicity through immune adjuvants. Su et al. constructed a multi‐adjuvant whole cell tumor vaccine by freezing method.^16^ With the action of cell‐penetrating peptide‐modified nanoparticles, interleukin 2 (IL‐2) and GM‐CSF were efficiently transported into cancer cells. After two cycles of freeze/thaw, the inactivated cancer cells with multi‐adjuvant as a cancer vaccine were successfully prepared. Serda et al. also designed a multi‐adjuvant autologous tumor cell vaccine for personalized therapy.^15^ As shown in Figure 14A, cancer cells obtained from mice or humans were suspended in a silicic acid solution at −80°C for 24 h. After coating Si cells with polyethyleneimine (PEI), pathogen‐associated molecular pattern (PAMP) CpG oligonucleotide (CpG ODN) and monophosphoryl lipid A (MPL) were decorated in the silicified surface. These pathogen‐mimicking cells could enhance tumor antigen uptake and presentation in vitro (Figure 14B,C). The silicified cancer vaccine can induce a protective response in vivo. In the mice with ovarian cancer, the syngeneic silicified cancer vaccine displayed the complete eradication of tumors and long‐term animal survival of mice (Figure 14D,E). The cryogenically silicified cancer cells functionalized with PAMPs provide a facile way to make individualized cancer vaccines with high efficacy.

Another way to improve the efficiency of cancer vaccines is through the combination of other treatments such as chemotherapy and photodynamic therapy (PDT).^66^ After the treatment of liquid nitrogen, Gu et al. suspended the inactivated tumor cells in the doxorubicin (DOX) solution to obtain therapeutics‐containing “dead cells” to treat acute myeloid leukemia (AML).^14^ Owing to the preservation of major structure and targeting capability, the obtained cells can enhance the targeting of DOX to the bone marrow. With the addition of an adjuvant, the DOX‐loaded whole cell cancer vaccine exerted synergistic effects of chemotherapy and immunotherapy. Besides, Li et al. designed an autologous tumor cell‐based vaccine hydrogel for tumor postoperative immunotherapy and PDT.^67^ Upon NIR laser irradiation, the hydrogel with the PEI‐Ce6 coated cancer cells inactivated by hypochlorous acid and freezing could activate DCs and mobilize cytotoxic T lymphocytes in the surgical region. The synergetic effects of cancer vaccine and PDT could efficiently inhibit tumor relapse.

## 6 SUMMARY AND PROSPECT

In summary, inactivated or living organism‐based therapy systems using the freezing method is a promising direction in biomedicine, including transplantation, tissue engineering, anti‐infection therapy, and anti‐tumor therapy. With the development of CPAs and temperature control technology, the cryopreservation of living organisms has become a routine procedure, and the related protocols have been standardized. Other than free organisms, microencapsulation‐ and scaffold‐based cell delivery systems can also be cryopreserved, which is beneficial to obtain standardized cell products on demand for transplantation and tissue engineering. Except for the storage of living organisms, the freezing method can impart therapeutic systems with splendid properties. The application of the freezing method during microneedle preparation or after traditional microneedle preparation allows the harvested vehicles to penetrate the skin easily while retaining the biological activity of the delivered organisms. These designs of cryoMNs and ice MNs further expand the application of freezing methods in living organism‐based therapy such as anti‐infection and anti‐tumor therapy. In addition to maintaining the viability of living organisms through cryopreservation, the inactivation of tumor cells by freezing is a simple and feasible method to prepare cancer vaccines. The combination of immune adjuvants or other treatment means could enhance the anti‐tumor efficiency of inactivated tumor cell‐based immunotherapy. All in all, freezing is a simple but viable method for both inactivated and living organism‐based therapy.

Although there are many advances, the clinical applications of freezing biological organisms still face some challenges. More preclinical studies and clinical trials are expected to evaluate the therapeutic efficacy of the tumor cells inactivated by the freezing method as cancer vaccines. For cryopreserved free organisms, higher viability is still an urgent problem. There are no standard protocols for the cryopreservation of the encapsulated cells. For scaffold‐based cell delivery systems, the non‐uniform distribution of CPAs and the compromised accuracy of cooling and warming need to be further explored. The large‐scale production of cryoMNs and ice MNs is a problem that needs to be solved. Based on the present research studies, we believe that the above problems will be solved in the future with the development of cryobiology, cryogenic technology, and material science. In conclusion, freezing biological organisms would continue to bring vast opportunities to biomedical fields and make contributions to human health.

## AUTHOR CONTRIBUTIONS

Yunru Yu conceived the conceptualization; Gaizhen Kuang wrote the paper; Qingfei Zhang, Jinxuan Jia and Yunru Yu contributed to the revision of the paper.

## CONFLICT OF INTEREST

The authors declare no conflict of interest.
